# Machine Learning for Analyzing Non-Countermeasure Factors Affecting Early Spread of COVID-19

**DOI:** 10.3390/ijerph18136750

**Published:** 2021-06-23

**Authors:** Vito Janko, Gašper Slapničar, Erik Dovgan, Nina Reščič, Tine Kolenik, Martin Gjoreski, Maj Smerkol, Matjaž Gams, Mitja Luštrek

**Affiliations:** Jožef Stefan Institute, 1000 Ljubljana, Slovenia; gasper.slapnicar@ijs.si (G.S.); erik.dovgan@ijs.si (E.D.); nina.rescic@ijs.si (N.R.); tine.kolenik@ijs.si (T.K.); martin.gjoreski@ijs.si (M.G.); maj.smerkol@ijs.si (M.S.); matjaz.gams@ijs.si (M.G.); mitja.lustrek@ijs.si (M.L.)

**Keywords:** COVID-19, machine learning, feature significance, feature correlation, risk factors

## Abstract

The COVID-19 pandemic affected the whole world, but not all countries were impacted equally. This opens the question of what factors can explain the initial faster spread in some countries compared to others. Many such factors are overshadowed by the effect of the countermeasures, so we studied the early phases of the infection when countermeasures had not yet taken place. We collected the most diverse dataset of potentially relevant factors and infection metrics to date for this task. Using it, we show the importance of different factors and factor categories as determined by both statistical methods and machine learning (ML) feature selection (FS) approaches. Factors related to culture (e.g., individualism, openness), development, and travel proved the most important. A more thorough factor analysis was then made using a novel rule discovery algorithm. We also show how interconnected these factors are and caution against relying on ML analysis in isolation. Importantly, we explore potential pitfalls found in the methodology of similar work and demonstrate their impact on COVID-19 data analysis. Our best models using the decision tree classifier can predict the infection class with roughly 80% accuracy.

## 1. Introduction

Coronavirus disease 2019 (COVID-19) is an infectious disease first identified in December 2019 in Wuhan, China. In most people, the disease is mild, but a fraction of the population—mostly older people with co-morbidities—experience severe respiratory symptoms, with cardiovascular and other complications. These lead to death in 0.7% of those infected [1], which is about 20 times worse than the seasonal flu. Because of the high mortality rate and infectiousness that is also worse than flu [2,3], strong countermeasures were adopted by most countries. Movement restrictions, temporary closure of businesses, and the like have led to severe economic activity and social life disruption.

The significant impact of COVID-19 on society resulted in substantial public interest. This in turn led to a quickly increasing body of research, ranging from forecasting the spread of the disease, diagnosis, risk assessment, and medication development, to—most relevant for our paper—the analysis of the impact of various factors on the spread of the infection [4,5].

After almost a year into the pandemic it is now clear that the strictness of applied countermeasures (e.g., school closing, mask usage, etc.) is the most important factor in determining the rate of the infection spread. However, there may be other important factors such as weather, air quality, population density etc. that also play a crucial role. To properly study the effect of these factors we analyzed the data from the early part of the pandemic when countermeasures were not yet applied.

Understanding different factors can help us clarify why the pandemic struck some countries more severely than others. In addition, in the case of factors that change with time, such as those related to the weather, it could help us predict which countries will be at the most significant risk and when. Finally, the impact of modifiable factors, such as those related to culture and human behavior, can be used to design more effective countermeasures.

The research analyzing the non-countermeasure factors that affect the spread of COVID-19 varies significantly. The papers investigate different countries, periods, infection metrics, and factors that affect the infection. However, a substantial fraction of the available work feels rushed and suffers from several weaknesses. First, many papers do not consider the low reliability of infection data due to insufficient testing in some countries. Second, the effect of countermeasures is not properly isolated from the effect of other factors. Third, the selection of the studied characteristics is incomplete, so an essential missing factor may be substituted for a correlated but unimportant one. This makes it difficult to uncover real causes for the severity of the infection. We attempt to address these weaknesses by studying countries and periods with sufficiently reliable data (based on their testing rate) before the countermeasures were implemented. We fuse many data sources (weather, demographic, economy, health, culture, travel, development) to create the most comprehensive set of potential country-wide infection factors to date. This data is analyzed with a range of statistical and ML methods to identify the factors affecting the spread of the disease as reliably as possible.

This paper’s key contributions are the careful selection of the countries, time intervals, infection classes for the analysis, the collection of the before-mentioned diverse dataset, and finally, we present a new algorithm for rule discovery. We mainly used classical machine learning (ML) algorithms but with carefully selected parameters and multi-step feature selection (FS). While classical in spirit, our methodology is thorough and, as a result, could provide the most reliable explanation for the early spread of COVID-19 infections to date.

**Figure 1:**
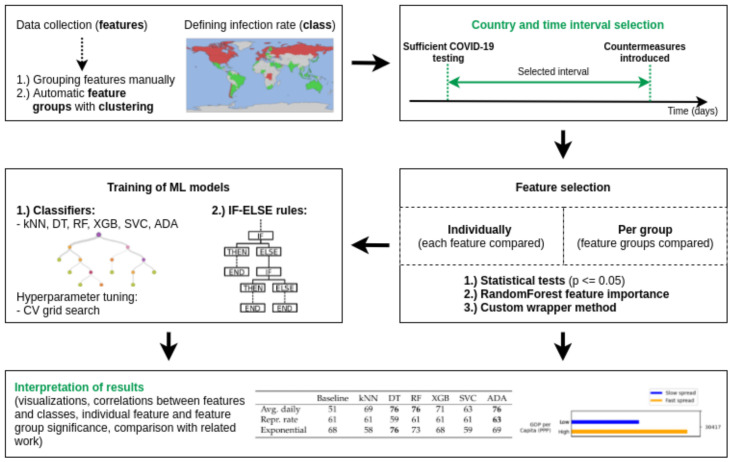
The overview of the research presented in this paper. Green titles indicate highlights of the presented research.

The rest of the paper is organized as follows. We start in Section 2 by reviewing the related work on the analysis of country properties affecting the spread of COVID-19, paying particular attention to its weaknesses. Then, in Section 3, we present how we collected our dataset. In particular, how to describe each country with different properties (features), how to decide on the time interval from which the data is taken, and how to transform the number of infections in each country to a binary infection class. This defines the problem setting of trying to understand which of the country properties most affect the infection class (is that country more or less severely affected by the disease). In Section 4 we then describe different methods—loosely grouped into two categories: statistical tests and machine learning—to achieve this task. A thorough examination of results (significance of individual features, as well as feature groups) is presented in Section 5. The paper finally concludes with a discussion of some of the more interesting features (Section 6) and general summary of our findings (Section 7). The described components of the paper are also shown in Figure 1.

## 2. Related Work

This section reviews the research that analyzes some properties of different world regions (typically countries) and compares them to a target variable related to the spread of COVID-19 in that region—with the goal of establishing the relationship between the two. The methods used range from classical statistical tests to various types of ML.

While the target variable was the spread of the disease in all cases, this spread can be characterized in many different ways. The number of daily infections was the easiest to monitor [6,7]. This approach is biased towards more populated countries, so an easy remedy is to consider the number of infections per capita [8]. Including the population as a variable in regression is also an option [9]. An alternative to looking at the number of infected was to look at the number of deaths instead [10,11]. This metric is more robust against under-reporting of cases (as deaths are more “visible”), but it can be heavily correlated with the health system’s quality. Another possible direction was to preprocess raw values before using them. The most common way of doing so was to calculate the *reproductive rate* [12,13] of the disease, which can approximate its spread speed and is often used in virology. Other options also exist; in the work of Cobb et al. [14], for example, they calculated the *compound growth rate*, while in the work of Lakshmi et al. [15], they calculate *virulency*.

Various potential factors (also referred to as features) have been considered for analysis. In some cases, the factor was known to affect similar diseases (e.g., the number of social contacts) and ML was used simply to confirm the hypothesis. In other studies, a large array of different features was used in an attempt to find new possible causes hidden in the available data. The most common features explored were based on the weather (e.g., temperature and humidity) [7,9,12,16,17,18], air quality (e.g., PM2.5—the concentration of particles under 2.5 μm in the air) [6,13,19], indicators of development, e.g., gross domestic product (GDP) [6,16], health care capabilities (e.g., the number of beds in intensive care hospital units) [7,10,20], population density [10,16], population age [14,15], interactions with other conditions (e.g., diabetes) [6,15,19], and social factors (e.g., social distancing, connectedness and mobility) [8,21,22]. Recent work by Duhon et al. [23] may be the most comprehensive with respect to the features. Data of these types exist in various public repositories and can thus be collected for many different countries.

The techniques used are just as varied. Classical regression methods were the most common [9,11,12,16,23], led by the simple linear regression. As with any domain nowadays, deep learning approaches were also investigated [24,25]. The next most common category was the statistical approaches [10,14], testing if any feature is statistically significantly connected to the class value. An unsupervised approach was used [18,26] to cluster the countries and then analyze the common properties of each cluster. In one case [15], the expert-based Total Interpretive Structural Modelling method was used in an attempt to determine the causal relationship between the variables. This is in contrast with the work whose main objective is accurate forecasting rather than determining the most predictive features. Such work is generally based on epidemiological models such as SEIR. The more sophisticated of these models also incorporate various features of countries they model, while the simpler ones just predict future infections from past infections. A review of models for multiple countries with a publicly available history of mortality forecasts [27] shows that the best of the models have a mean percentage error of 7%, 14%, and 24% when forecasting 4, 8, and 12 weeks ahead from July 2020.

In most cases, the authors of the related work show that their selected feature is correlated to a COVID-19 infection metric, and every previously mentioned feature is considered significant in at least one of the studies. This could indicate that the rate of infection is based on a variety of different factors. While some studies agree on the significance of features, some are still wildly disputed. For weather, for example, it is still unclear whether it affects COVID-19 infection, with several published papers claiming either that it does [7,12,28] or it does not [29,30,31]. A recent investigation of this question finds that the the answer is inconclusive [9].

The plethora of different conclusions could also mean the methodology used for determining them is not stringent enough—common problems found in the literature are thus addressed in the next section.

### 2.1. Common Methodology Weaknesses

Given the desirability of fast results in this domain, much of the published research exhibits methodological problems. In this section, we try to outline these problems and how we tried to avoid them in our paper. This section can provide insight into how our work compares to others and a potential guideline for future researchers in the same domain.

#### 2.1.1. Ignoring Testing

Studying the disease in its early phase has its upsides and downsides. On the one hand, there are no countermeasures present, which allows us to observe how the disease behaves when uninhibited. On the other hand, the data in that period is the most lacking and inconsistent. The volume of testing for COVID-19, in particular, was considerably different across different countries.

Not adjusting for the testing rate can seriously skew the research results. In particular, many countries had the testing rate much below the recommended threshold and thus should be excluded from the analysis. To illustrate the danger of not doing so, we show in Section 5.3 that many features are statistically significantly connected to the infection rate when all countries are considered, but the connection disappears when only the countries with the appropriate amount of testing are analyzed.

The most obvious example is that testing was scarce in less developed countries; thus, no COVID-19 infections were found. Either ML or statistical models can then learn that any feature connected to low development also inhibits the infection rate. While this is plausible for some features (e.g., low urbanization), it is improbable for others (e.g., low quality of healthcare).

To reduce the problem, we excluded every country with insufficient testing, and for every other country only took data starting from the period when sufficient testing was conducted (Section 3.2). In addition, some of our classes derived from the infection rate were partially resistant to the lack of testing (Section 3.1).

There are some valid alternatives to our approach. The first is trying to correct the lackluster data, for example, by considering the death rate as well as the infection rate [32,33]. The second is to have the testing rate explicitly coded in the analysis, for example, as a feature in ML [26]. In some cases, the problem of testing was partially solved implicitly by working only on a small subset of hand-picked usually developed and well-tested countries [10,17,22].

#### 2.1.2. Ignoring Countermeasures

Any well-designed countermeasure will obviously greatly reduce the spread of the infection. This was empirically validated in Section 5.1 by showing that for some classes, the infection rate quickly changed after the measures were taken. Nonetheless, they are rarely explicitly considered in the analyses done by the related work.

In almost all cases, authors chose an arbitrary endpoint (usually in early April) and took data only until that point. Duhon et el. [23] used the peak of the first wave as the endpoint, which may be preferable as it is adapted to each country. However, both approaches can be considered inadequate as countermeasures were established at different points in time.

In our work we took the countermeasure data for each country, which helped us better define the correct end points when countermeasures started for the analysis for each country (Section 3.2). As with the testing, a valid alternative is to use the countermeasure data as a feature when modeling the infection rate to avoid discarding the data [10,16].

#### 2.1.3. Limited Data and Misleading Conclusions

In a substantial part of the reviewed work, the authors focus only on a small number of features or on the features all from the same category (e.g., development, weather, health-care capacity, etc.) This is not necessarily a flaw as it allows for a greater depth of analysis of that particular data type.

However, in this particular domain there are many different features from many different categories that are correlated with the COVID-19 spread. By only considering a few of them, one cannot determine which ones have the most significant impact or which actually cause fast infection spread rather than are merely correlated with it. For example, it has been shown [6] that GDP is correlated with the infection, but it is almost certain that it is not causing it directly. It is more likely that larger GDP implies, for example, larger cities or more tourists and, by proxy, more social contacts, and thus more infections. So it is preferable to not only have GDP as a feature but also features characterizing population density and tourism.

Having many features is not enough, especially in cases where causation is less clear. For example, in theory, the high temperature could affect the spread of COVID-19 (as it does with some other diseases). However, it could be merely correlated with GDP, as most countries with a high GDP (which tend to be highly infected, or at least have sufficient testing to detect this) have a similar continental climate and thus similar temperatures. Unfortunately, ML does not provide a good way of differentiating between these two cases, which is a limitation infrequently discussed in the related work.

While all our data is collected from publicly available sources, we have to the best of our knowledge collected the most exhaustive dataset of different features and classes. Our dataset is also publicly available for other researchers of the domain to use. This is, in many cases, not enough to make conclusions about causation though. Like the related work, we cannot truly overcome this limitation, but we mitigate it by exploring and presenting correlations between features, thus allowing health experts, policy makers, and other researchers to make use of our results.

## 3. Dataset

This work aimed to determine if conditions in a given country before any countermeasures are taken were suitable for the fast spread of COVID-19. In the following sections we define the metrics used for measuring the infection rate as well as different features that were considered as potentially correlated with the said rate.

All data was collected on a per-country basis. While the infection data is frequently available on a regional basis, most of the feature data we collected is not. Thus, although the regional analysis would offer greater precision, we opted against it.

In ML terminology, every country became one instance. Different factors that could affect the spread of the infection (Section 3.3) were the features for that instance while the binarized infection-rate metric became its class. Different infection metrics were tried, as described in Section 3.1. This dataset is publicly available and described in detail in [34].

### 3.1. Classes

Determining if a region is “suitable” for the virus’s fast spread is not trivial even from a time series of infections. Many approaches have already been tried, and some of them are described in the related work (Section 2).

We calculated several of the proposed metrics and realized that while many are highly correlated between themselves, others are surprisingly not—they seem to model different notions of “fast spread”. We chose three distinct metrics described in the following sections. When describing a metric, we also briefly mention the highly correlated alternatives.

In all cases, we decided to binarize the metric into two classes: a country is considered positive if its infection rate, given the metric, is faster than roughly half the countries analyzed. This simplification was made because—given the incompleteness of the data—it is sometimes hard to determine the exact number of infected, but it should at least be possible to decide if the country is one that is more prone to infection or not.

The time series analyzed regarding the infection rate was taken only from a specific time interval that was different for each country. The exact time interval is described in Section 3.2 and can be roughly described as starting when testing was adequate in a country and ending when countermeasures were applied.

#### 3.1.1. Daily Number of Infections

The first calculated metric was the daily number of infections, averaged over the appropriate time interval and normalized based on the country population. While crude, this metric is the one usually reported by the media and can be considered the most intuitive. While it does not tell much about the future speed of infection growth, it can be indicative of the current situation.

A similar metric calculated, but not used, was the absolute number of infections, normalized by the population, on the last day of the time interval. While this metric conveys similar information, it is more heavily influenced by the day the countermeasures start—as that defines the end of the time interval. Still, the two metrics have a Pearson correlation coefficient of 0.85.

#### 3.1.2. Reproductive Rate

The reproductive rate $R_{0}$ [35] is a metric commonly used by virologists to determine the severity of an infection. Simply put, it estimates how many people are infected by each currently infected person. If $R_{0}<1$, the infection should subside in time; if $R_{0}>1$, however, the infection is predicted to spread exponentially.

To estimate the reproductive rate, we used the SIR model [36]. This model assumes that the population is divided into three groups: susceptible *S*, infected *I*, and removed/recovered *R*. At each time step, αSI of the population goes from the susceptible group to the infected one. It is assumed that every infected individual can contact every susceptible one, hence SI possible interactions. In addition, in each time step, βI individuals go from the infected to the recovered/removed group. After estimating the parameters α and β, the reproductive rate is given as $R_{0}=\frac{\alpha S(0)}{\beta }$.

Aside from the reproductive rate, there are other metrics that can show the acceleration of infection growth. We also calculated the doubling rate, i.e., the average number of days it takes to double the number of cases. Interestingly, the countries considered “positive” using this class were exactly the same as those considered positive with the reproductive rate metric. Hence, these two could be used interchangeably on this dataset. The second alternative tried was the ratio between the number of infected this week and the number of infected in the previous two weeks (those are still potentially infecting others). This ratio gives a simple estimation of $R_{0}$ and it indeed showed a correlation of 0.86 with the $R_{0}$ calculated using the SIR model.

#### 3.1.3. Exponential Shape

The last metric we calculated was the shape of the infection time series. An exponential shape visually and intuitively indicates that the number of infections is rising fast and is likely to do the same in the future. Alternatively, if the growth is linear, one could assume that the situation is more “under control”. Another way of looking at it is that according to the infection models (like the *SIR* model), the number of infected grows exponentially—if it does not, something could be inhibiting its growth. Aside from linear and exponential functions, one can also find attempts at fitting more complicated functions [37], but we were mainly interested in the exponential nature of growth.

To determine if the growth is exponential, we fitted both a linear (y=ax+b) and an exponential curve ($y=ae^{bx}$) to the data—the number of daily infections for the given time interval. The parameters *a* and *b* were in both cases determined so as to minimize the mean square error of the approximation. After both were fitted, the one with the lower error was chosen as the better fit. If the exponential fit was better, the class value for this metric was positive.

It is of note that this metric and the previous one (reproductive rate) are partially resilient to the lack of testing. For example, if only each 10th infection is detected, the data’s overall shape and exponentiality remain the same. This relies on the assumption that the amount of testing is constant and that there is no bias in the procedure, which may, of course, not be (entirely) true in reality.

As an alternative, we can consider that in most cases, the infection can spread from an individual to others before they are officially confirmed infected. Thus the exponential term could be based on the past number of cases. In this case, the exponential curve could have the form of: y(i+1)=y(i)+ay(i−k), where *k* is the average number of days from infection to being confirmed. Empirically, however, this model produced a less accurate fit to the data, so the base model was used instead.

#### 3.1.4. Class Comparison

**Figure 2:**
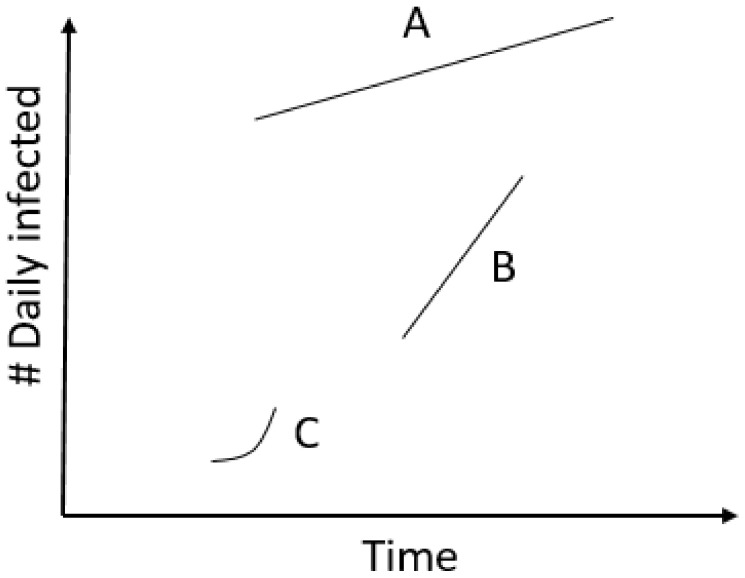
Hypothetical infection curves. Curve A represents a sample positive only according to the *Avg. daily infections* metric. Curve B is positive only according to the *Reproductive rate*, and curve C only according to the *Exponential* metric.

The *Avg. daily infections* metric mostly indicates the current situation in the country, while the *Reproductive rate* metric, if positive, represents that the situation is worsening fast. The *Exponential* metric, on the other hand, is not interested in either the current situation or the current rate of change, merely that the change is exponential. While most countries that are classified as positive are positive according to more than one metric, we show sample curves of those that are positive with only one (Figure 2).

**Figure 3:**
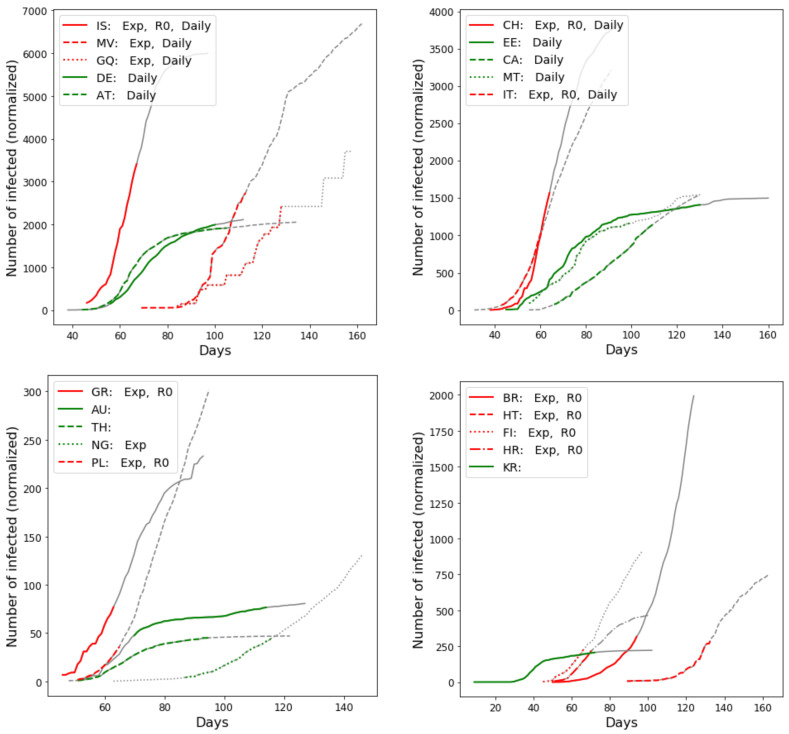
A sample of 20 curves, each representing infection progress in a different country. The legend shows the ISO code of the country and which classes are positive. A line is red if at least two out of three metrics label the country as positive. Gray lines indicate the part of the time series outside the time interval used for our analysis.

Actual curves of total numbers of infected for some sample countries are provided in Figure 3. The same figure also shows to which classes a curve was classified into, to illustrate the difference between them.

### 3.2. Country and Time Selection

An important contribution of this paper is determining which countries are suitable for this analysis, and for each country, which time interval to use. The time period had to have two desirable properties: (a) the testing should be at least adequate and (b) countermeasures should not yet be in effect.

The World Health Organization (WHO) recommends that less than 3–10% of all tests made are positive [38]. This ensures that the testing is widespread enough and not done only on the most severe cases, ignoring the larger portion of (currently) less severe cases. While we started with this recommended threshold, we had to decrease it to 15%, as in the earliest stages, before widespread countermeasures, testing rarely achieved the ideal rate. This rate determines, for each country, when to start calculating the class.

To limit the time interval in the other direction, we estimated when at least moderately strong countermeasures were implemented for each country. We did so by finding the first date on which there were at least three different countermeasures implemented, based on a public data repository [39]. The decision to require more than one countermeasure stems from the fact that the earliest countermeasures were limited in scope (e.g., banning of concerts) and probably not effective enough. We delayed the countermeasure start by one week, as any new policy has an effect on the confirmed cases with the delay of at least the disease’s incubation period [40].

#### 3.2.1. Selection Procedure

The interval selection step by step:

- Having the start and end date for each country, as defined by the testing rate and countermeasures, we selected the countries for which these dates form an interval. In some cases, the interval cannot be formed because a good testing policy was never established (no start date), countermeasures were never established (no end date), or they came earlier than good testing (end date before the start date).
- For the selected countries, we calculated their average duration and the average number of infections at the start of the interval. In addition, we defined each class as being positive if the corresponding metric (Section 3.1) exceeds its median value—this threshold value was also stored. This way, half the countries would be considered positive and half negative. The exception to this rule was the *Exponential* class, which did not use any threshold value but simply compared if the exponential fit was better than the linear one.
- Countries with no end date defined by the countermeasure start were added to the list of selected countries, and their interval length was defined by the average interval length calculated in the previous step. This ensured that these countries have a similar interval lengths as the others.
- For each country with no start date defined by testing (or if testing came too late), we created an interval that started on the day when the number of infections matched the average starting number of infections in other selected countries. Classes were defined using the precalculated threshold values for each metric, and using them, we calculated the majority class over the three class definitions. If the majority class was positive, this country was selected for further analysis; otherwise, it was rejected. The logic goes as follows: if there was no adequate testing on an interval, but there was still enough infection for the class to be positive, it is reasonable to assume that the class would be positive with more testing and thus more infected people would be found. On the other hand, if we have inadequate testing and negative class, we cannot say anything reliable about the infection in that country.
- The rest of the time intervals (and corresponding countries) were excluded from the list of selections.

The advantage of our country and time selection over less strict or more ad hoc selections is (1) that the data on infections is more accurate and, more importantly, comparable and (2) that the impact of countermeasures is largely excluded, allowing other factors to show. The disadvantage is that we had to discard the data from some countries entirely.

**Figure 4:**
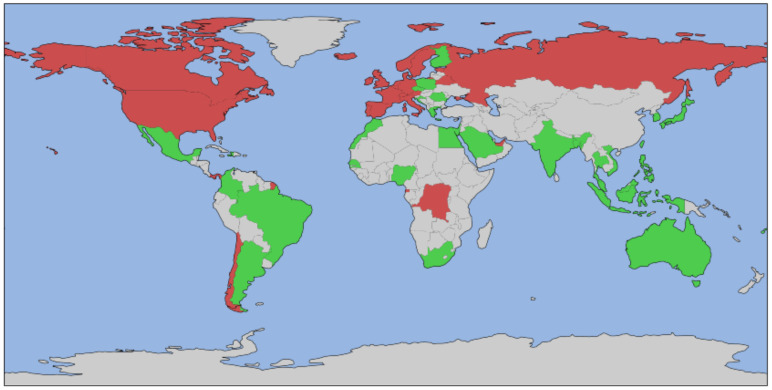

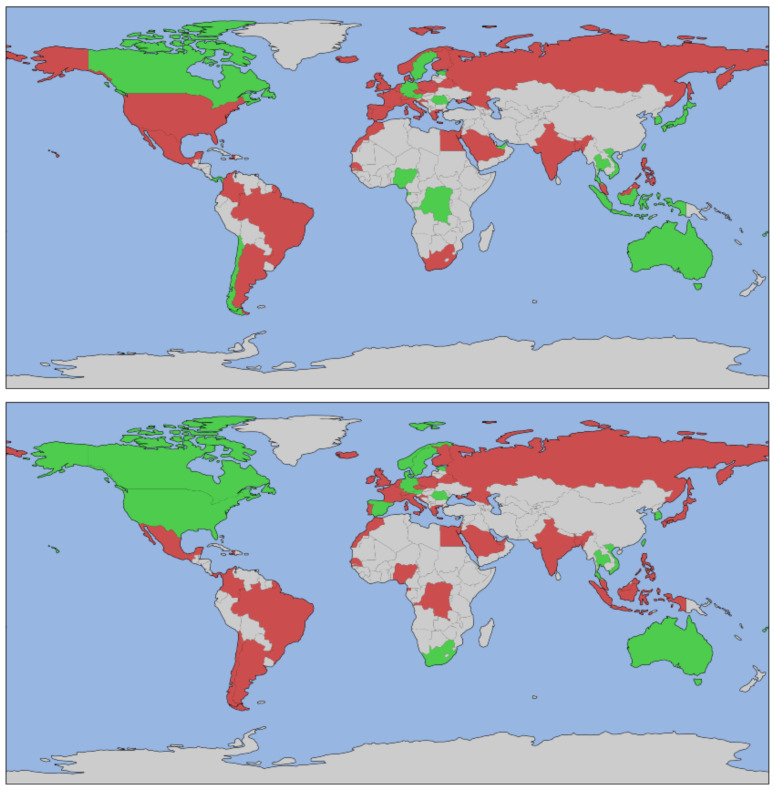
Countries were painted red if considered infected, green if not, and gray if the country was excluded from the analysis. **Top**: *Daily avg. infections*, **Middle**: *Reproductive rate*, **Bottom**: *Exponential*.

In Figure 4 we show every country colored based on whether it was selected for the analysis and based on the value of each binary class.

#### 3.2.2. Alternative Country Sets

By and large, we used the previously defined country set (denoted as *Selected*). However, to show the effect of a different country selection, we defined a few alternative country sets.

The first alternative set (*All*) contained all the countries for which at least 70% of feature data was available. The missing values were then filled using an Iterative imputer—a multivariate imputation approach that estimates each missing feature value from all the other known feature values. Some largely similar countries can differ in some features, and the Iterative imputation method can learn such complex relationships between features (while Nearest-neighbour and some other imputation methods do not).

The second (*Developed*) contained only countries considered developed [41]. For a specific test (Section 5.5) we devised two additional sets: *Semi-developed* contained half the developed countries and the same number of nondeveloped countries (both randomly chosen), while *Semi-selected* contained the same balanced mix of selected and not-selected countries. This choice was made so we could validate if the classification difficulty arises from the set size or from the type of countries in it. For each of these sets, we report their size and the proportion of instances classified as positive for each class in Table 1. It can be observed that the choice of the countries and the class has a significant effect on the proportion of the positive class (fast spread of infection).

### 3.3. Features

Features for this research were obtained from publicly available databases to guarantee transparency and replicability. Altogether, 135 features were collected and described in following subsections (full list in also Section 5.1). Any custom preprocessing used on these features is also described here.

#### 3.3.1. Weather

The weather data [42] in our features includes data on humidity, maximum, average (with average low and high daily temperature), minimum temperature, apparent temperature, cloud cover, climate (based on biogeographical and development criteria from [43]), dew point, ozone, PM2.5 particle concentration, precipitation intensity and probability, air pressure, ultraviolet (UV) index, visibility, wind speed, and gust. Daily measurements were averaged over the same time interval from which the infection data was taken (different for each country).

#### 3.3.2. Culture

The features that were categorized as “Culture” come from four sources: the Big Five personality traits (B5) theory [44], Hofstede’s cultural dimensions theory [45], research on human spatial behavior [46], and research on cultural tightness [22]. This data was included to offer novel insights into the spread of COVID-19 in terms of human social behavior as well as to include data from a domain not usually considered in similar research.

For the purposes of this work, the answers from 1,015,342 subjects to the B5 questionnaire [44] were analyzed by calculating the scores on the B5 dimensions. Afterwards, the subjects were grouped by their country of residence, and the mean scores for each trait were calculated for each country, thus creating a novel psychological construct of country psychological traits as opposed to subject-only psychology traits. For reliable results, only the countries with equal to or more than 100 subjects that answered B5 were used. Next, the features were normalized by transforming scores into percentiles.

Individualism-collectivism describes how much a society’s members prefer to form in-groups and how strong these are. Uncertainty avoidance describes how averse a society’s people are to uncertain events. Long-term orientation describes how traditional a society is and how much it embraces adaptation and progress—the higher the score, the more long-term the orientation. Power distance describes the strength of social hierarchy or the lower strata’s perception of how equally the power is distributed in society. Indulgence describes the degree of freedom people have in fulfilling their desires. Task- vs. person-orientation describes a society’s preference towards tasks (achievement, assertiveness, material rewards) versus people (caring for the weak, cooperation, modesty).

Two more features belong to the “Culture” category: Social distance describes the preferred physical interpersonal distances [46]. Tightness describes the strength of social norms in a society [22].

#### 3.3.3. Travel

The chance of the virus spreading through the country or area is closely connected with the volume of foreign and domestic passengers and tourists [47]. To account for this, two datasets for passengers’ volumes by country were used [48,49]. The most current data was used. The data was normalized so it is easier to compare; the normalization was done by max value in both datasets. The datasets’ features include the number of plane passengers, the number of tourists, net migration, driving mobility, walking mobility, foreign direct investments (FDI) data (investments made by one entity from one country into another located in another country), and GDP.

Mobility information was obtained from the COVID-19 mobility trends dataset [50], made available by Apple, who obtained it from people using their devices. Data contains a relative volume of directions requests in the Apple Maps application per country/region, subregion, or city compared to a baseline volume on 13 January 2020.

#### 3.3.4. Health

It was consistently observed that older patients with co-morbidities and lifestyle risk factors are affected by COVID-19 more strongly [51,52]. These risk factors affect the severity of the disease, while it is not clear if they also affect susceptibility and infectiousness. Symptomatic carriers are more infectious than asymptomatic ones, though [53]. We included the prevalence of the chronic diseases with the best evidence for impact on COVID-19: diabetes, cardiovascular disease, respiratory disease, chronic kidney disease, dementia, and cancer [54]. The prevalence of obesity [55] and smoking [56] were included as the main lifestyle risk factors. The median age [57], birthrate, and death rate [58] were used to characterize the population’s age structure.

ACE receptor–a protein on the surface of many human cell types—serves as the entry point for the SARS-CoV-2 virus causing COVID-19. There are two main variants of the gene coding for this protein: insertion (I) and deletion (D). The country-wise frequency of the D variant was shown to be correlated with the COVID-19 prevalence and mortality by Delanghe et al. [59]. We used as a feature the related frequency of the II combination [60], with insertion on both chromosomes, which was the subject of an earlier version of the Delanghe et al. paper. Blood type was also found to be associated with COVID-19 infection [61]. Blood group O was most consistently depleted among infected people, so we included its country-wise frequency [62] among the features. Since the data for these two features is sparse, and they are determined genetically, we estimated the missing values by using the average value of the three genetically closest countries [63], weighted by the inverse genetic distance.

There is some evidence that vitamin D may help fight COVID-19 [64]. Vitamin D deficiency is widely studied, so we used the country-wise prevalence of vitamin D deficiency, defined as blood level of <50 nmol/L, as a feature [65]. The average prevalence for adults and seniors was used where available. The Bacillus Calmette–Guérin (BCG) vaccine against tuberculosis confers broad protection against infectious diseases, and there is some evidence it also helps against COVID-19 [66]. We thus added the BCG immunization percentage to the features [67]. Since several countries discontinued immunization, the last available immunization percentage was used, decreased by the birthrate for every year of nonimmunization.

#### 3.3.5. Economy

Features on the economy were collected from three databases: ArcGIS World GeoEnrichment Service [58], Countries of the World [68], and The World Bank [48,49]. The features include GDP, unemployment, personal and corporate taxes, tariffs, public debt, fiscal health, inflation, and government spending.

#### 3.3.6. Development

The category of “Development” uses multiple data sources [48,49,58,68] to describe various dimensions of progress and development of a country. While many depend on the economy, we excluded direct economic features, as the previous category addresses those. The features include data on whether the country is considered developed by certain sources, overall country, region, and world prosperity scores, number of phones per 1000 people, literacy percentage, quality of education, infant mortality, government efficiency, government integrity, the contribution of agriculture, industry, and service to GDP, monetary freedom, labor freedom, financial freedom, trade freedom, property rights, business freedom, and judicial effectiveness.

#### 3.3.7. Geography

The features under the category of “Geography” mostly consist of a one-hot encoded region of the world [68], alongside data [58,68] on area, whole and urban populations, population density, the ratio between area and coastline, percentage of arable land, and percentage of arable land used to grow crops.

#### 3.3.8. Countermeasures

To account for countermeasures in our analysis, we included two features: eventual countermeasures [69], which is a binary description of whether a country has implemented countermeasures by a specific point in time, and COVID awareness, which tracks Google trends in researching COVID-19 [70].

## 4. Method

Our analysis had four major components. First, we verified the significance of different features, both individually (Section 4.1) and combined into different feature groups (Section 4.2). Second, we created ML models that try to use these features to make predictions on which countries were most vulnerable to infections (Section 4.3). Third, we present a rule-discovery algorithm that can explain the best performing models, illustrating the connection between the features and classes (Section 4.4). Last, along the way of the previous three points, we showcase both the effect of common pitfalls found in the literature and the limitations of the pure ML approach.

### 4.1. Feature Significance

The primary goal was to find features that indicate why early infection spread in some regions was faster than in others. The first step for doing so was finding the features correlated with one of the three selected classes. The strength of this correlation was first tested by using statistical significance tests on both features and feature groups. Then, we used a ML metric *Random Forest (RF) feature importance* to tackle the problem from a different angle. Finally, we validated the usefulness of different feature groups by using them to create accurate ML models (this last approach is described in more detail in Section 4.3).

#### 4.1.1. Statistical Significance

For statistical testing we first had to divide features into four groups—*continuous normal*, *continuous non-normal*, *discrete binary*, and *discrete categorical*—as we had to use different statistical methods for each feature type. To determine which continuous variables are normal and which are not, we ran the Shapiro–Wilk normality test. We set the threshold for *p*-value at 0.05.

Once the features were grouped, we ran the statistical tests for all three classes (*Daily avg. infections*, *Reproductive rate* and, *Exponential*) on three datasets (*Selected*, *All*, and *Developed*).

**Figure 5:**
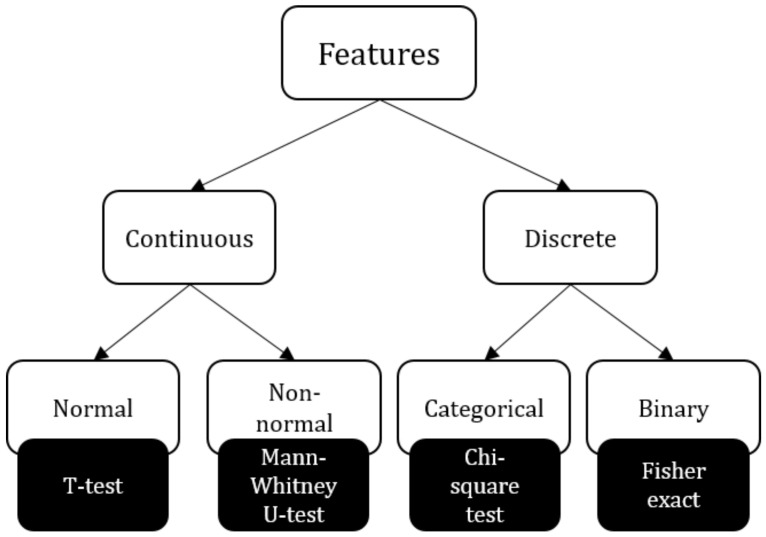
Statistical tests for each feature type.

We compared the feature values of countries positive with respect to a class to those negative with respect to a class. We used the *T*-test, Mann–Whitney *U*-test, Chi-square test, and Fisher-exact test, respectively, for continuous normal features, continuous non-normal features, categorical, and binary features (see Figure 5).

The statistical tests returned *p*-values that give information about the feature significance. We set the significance threshold at p=0.05. As we ran a large number of statistical tests, we can expect that some of the features were found as significant purely by chance. Therefore, we used the multiple comparison method (step-down method using Bonferroni adjustments) to correct the *p*-values. This correction is not usually done in the related work but has a significant impact on the results. These values are listed as “corrected” in Section 5.3.

#### 4.1.2. Ml Significance

Several metrics are used to rank features in ML, like *Information Gain* or *chi2*, out of which we opted to use the *RF Feature Importance*. In summary, this metric trains a RF classifier consisting of several different trees. When training a tree, it computes how much each feature decreases the weighted impurity in this tree. This impurity decrease is then summed up over all the trees in the forest to form the feature importance.

One of the advantages of using this method is that it implicitly accounts for the features that are both mutually correlated and correlated with the class. *Information Gain*, for example, would put all such features at the top of the list, despite the fact that they are mutually redundant. This in turn would cause a FS method that takes the top *n* features to take only very similar features (of which there are many in our dataset).

In the case of calculating the importance of a feature group, we simply summed the importance of all its members. This does somewhat favor larger feature groups. Of course, larger groups may indeed contain more information, and this is why we opted for the *sum* and not *mean* instead.

### 4.2. Creating Feature Categories

We investigated the existence and importance of potentially semantic feature groups within our collected data. We hypothesized that groups of features generally describe the same country property and thus carry similar information.

#### 4.2.1. Manual Categorizing

Our initial approach was to inspect all our features and manually determine semantic clusters by placing each feature into a category. This resulted in the feature categories given in Section 5.2, where some features were also excluded due to high internal correlation with others in the same category. More details about the features and their categories are given in Section 3.3.

#### 4.2.2. K-Means Clustering

Since our manual categorization was based on our understanding of the features and thus subjective, we decided to investigate some clustering algorithms, which use similarities or differences between features that may not be apparent to humans. Initially, we used one of the most widely accepted and tested algorithms, which is K-means clustering. We used the default implementation from scikit-learn [71], which attempts to minimize inertia or within-cluster sum-of-squares criterion.

The key parameter to K-means is *K*, which specifies the number of clusters in advance. As our goal was to step away from human influence, we did not choose *K* as the number of clusters in manual clustering but instead used the silhouette score to determine the optimal number of clusters from the data. The silhouette score is a measure of how similar an object (in our case, a feature) is to its cluster (cohesion) compared to other clusters (separation) and can take values from −1 to +1, where a high value indicates that the object is well matched to its cluster and poorly matched to neighboring clusters. We investigated *K* in the range of [2, 15] and found the highest silhouette score is achieved when K=4 (features in these clusters are listed in Table A1).

Usually, clustering is used to find subgroups of similar instances; however, in our case, we wanted to find groups of similar features instead. Thus, the input to the K-means algorithm was a transposed input matrix, where rows were features and columns were countries.

Once the clusters of features were obtained, we used a dimensionality reduction technique to obtain a single artificially created feature that best describes each cluster. To do this, we used Principal Component Analysis (PCA) across all the features within each cluster, reducing the dimensionality to a single dimension. The aim of getting this single feature was to evaluate the correlation between different clusters and to use it for computation of statistical significance of each cluster.

#### 4.2.3. Hierarchical Clustering

In addition to K-means, we also tried hierarchical clustering. Initially, we used the same approach as previously, inputting the transposed data matrix, which results in computing the Euclidean distances between features. As an alternative, we also used a custom distance function where the distance between two features was defined as the absolute value of the correlation between them.

**Figure A1:**
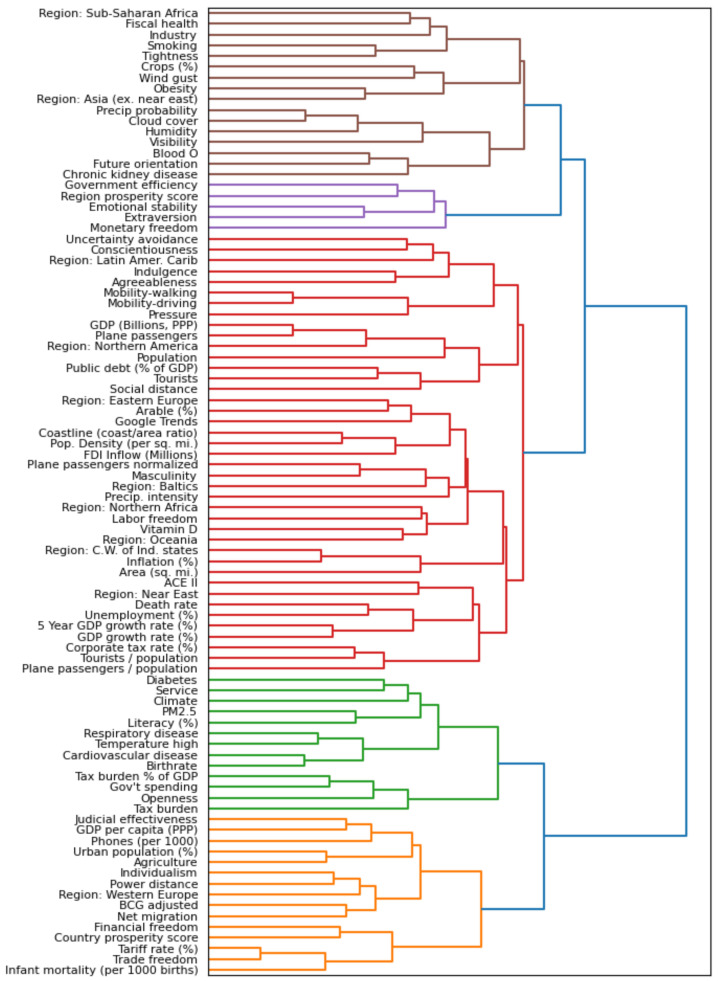
A dendrogram of features, connected based on the strength of their correlation, created with hierarchical clustering.

In this case, we chose the number of clusters as 8, matching the number of manual categories. A dendrogram that shows how other cut-off points would look is shown in Figure A1. Similar to K-means, we again computed the single PCA-reduced feature to use in inter-cluster correlation and statistical significance analysis.

### 4.3. Classification

As the ultimate aim of most work dealing with COVID-19 is to investigate what influences the virus’s initial spread, we also investigated some predictive models to see how successful such predictions can be. Unlike most related work, we formulated our task as a classification problem, defining binary classes related to the virus’s spread, as described in Section 3.1.

#### 4.3.1. Algorithms

Once the class and the full set of features were chosen, we chose a set of classification algorithms to investigate. The set includes k-nearest neighbours (kNN), Decision Trees (DT), Random Forest (RF), Extreme Gradient Boosting (XGB), Support Vector Machines for Classification (SVC), and AdaBoost (ADA). All the models trained with these algorithms were always compared against a baseline classifier, which predicts the majority class.

#### 4.3.2. Evaluation Scheme

We chose a leave-one-instance-out (LOIO) evaluation scheme, in which we always trained our model on all instances (countries) except one and tested on the one left out. This was repeated *n* times, where *n* was the number of instances, ensuring that each instance was classified once. The accuracy was the average over all the iterations. Such a schema is very robust due to several reasons. First, it naturally ensures that each instance that is being tested is not seen during training. Second, it always leaves enough instances to train an informative model, even when the number of instances is low—which was a problem in our case, especially when subsets of all data were used. Most importantly, LOIO does not suffer from the volatility resulting from a specific (random) train-test split, as we observed that the results can vary greatly between different splits on our data.

#### 4.3.3. Data and Feature Selection

Three different sets of instances described in Section 3.2—*All*, *Selected*, *Developed*—were used for evaluation. We investigated all features as well as subsets of features obtained with different FS methods and repeated the experiments for each of the three classes (that describe the virus’s spread) given in Section 3.1.

We investigated all features as well as subsets of features obtained with different FS methods and repeated the experiments for each of the three classes given in Section 3.1. In the initial setup, we used the full feature set; however, the number of features exceeded the number of instances, potentially causing over-fitting. In addition, many features may be redundant or not informative. Therefore, we investigated three FS procedures:

1. **RF feature importance**: We sorted the features based on this metric and then took the best 20 features for the classification. This value was chosen as lower values (10 or less) did not achieve high classification accuracy.
2. **FS with a Wrapper method:** In addition to using the previously mentioned out-of-the-box RF feature selection, we also investigated a custom FS algorithm similar to the one used in our related work [72]. First, the features were sorted using RF feature importance as before. Then, if two features were correlated (Pearson coefficient >0.7), we discarded the lower-ranking one. We started by using only the best feature for the classification. Then, we iteratively added the next best one but only kept it if it did not decrease the classification accuracy by more than two percentage points. To determine the classification accuracy, the whole LOIO procedure was repeated. This Wrapper procedure was then repeated with the next best feature, etc. The whole experiment was also conducted with slightly different threshold values, but by doing so, we achieved very similar results.
3. **Boruta FS**: A publicly available FS method that tries to isolate all nonredundant features [73]. This FS method was tried as it was used in another COVID-19 related work [74].

#### 4.3.4. Hyper-Parameter Tuning

Most classification algorithms offer a variety of hyper-parameters to be set, which can notably influence the performance. Due to this, we investigated a range of hyper-parameter values for all the used classifiers using a 5-fold-cross-validation (CV) grid search. This method evaluates a predefined grid of hyper-parameter values by doing a CV for each possible set of values from the grid. It then chooses the set of hyper-parameter values that achieved the best result in the CV according to a predefined metric—in our case, this was the accuracy.

### 4.4. Generated Rules

To gain an insight into models that classify countries into those more and less prone to the fast spread of COVID-19, we designed a problem-independent rule learning algorithm. The input to the algorithm is preprocessed by discretizing features into low and high values, where the split between the two is done so that the resulting two groups of instances have as low-class entropy as possible.

The rule learning procedure evaluates all possible rules with respect to the discretized features. Our rationale was that we wanted to make sure all possible relations in the data were considered and none was left out because of a bias in the learning procedure. When applied to the domain, particular relations of interest can then be investigated in more detail, while other relations deemed coincidental by the domain knowledge can be discarded. The algorithm is a successor of the HMDM algorithm [75], where the emphasis is on designing a tool that helps human analysis. Compared to the out-of-the-box alternatives, our algorithm creates a more complete overview but is more computationally expensive.

There are three options for each feature: the feature is not in the rule or the feature is in the rule and is equal to the low or high value. This results in $3^{n}$ rules to be evaluated, where *n* is the number of features. Thus, it was not feasible to use this procedure on all the features, so we used it on features selected with the Wrapper method. Three metrics are calculated for each rule: (1) coverage, (2) accuracy on covered examples, and (3) rule score as coverage multiplied by accuracy. Rules with low coverage or low rule score on the training set are discarded. The remaining rules are sorted with respect to the rule score, and the best 1000 rules per each class value are retained.

The obtained set of rules is used to assess the relation between discretized feature values and the class value. A feature value $f_{i}$ contributes to a class value *c* (being slow of fast spread of COVID-19) if adding the ${\mathrm{feature}}_{i}=f_{i}$ term to a rule of the form:

$$\mathrm{IF}{\mathrm{feature}}_{j}=f_{j}\mathrm{AND}{\mathrm{feature}}_{k}=f_{k}\mathrm{THEN}\mathrm{class}=c$$

increases the coverage-multiplied-by-accuracy rule score. The rule score increment or decrement represents the feature score. Rules where feature values make negative contributions (i.e., those with negative feature scores) are discarded, and the feature scores of each feature value are summed up across all the rules for each class value.

**Figure 6:**
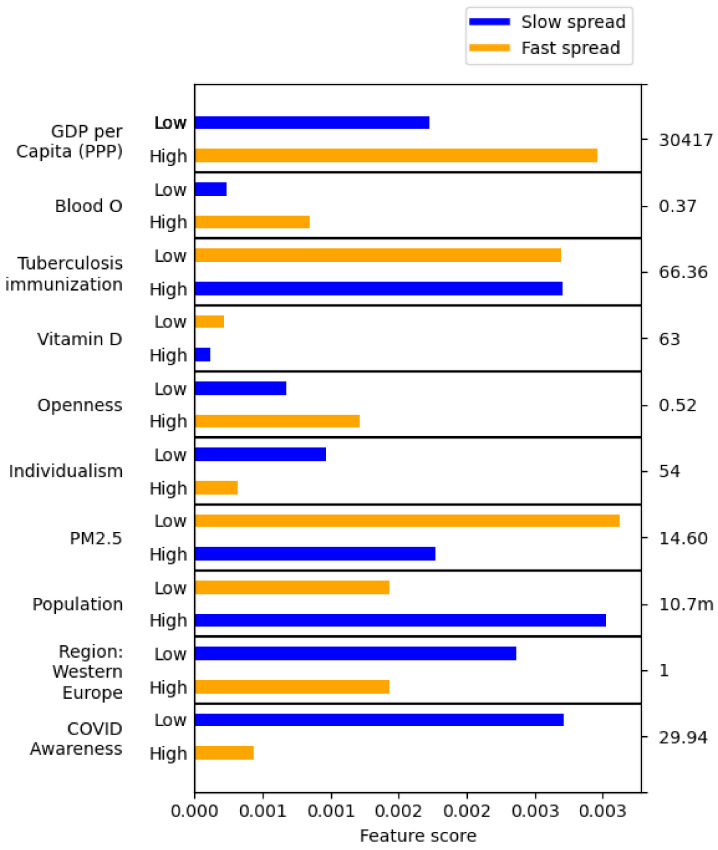
The contributions of feature values discretized into low and high to the slow and fast infection spread for features selected using the Wrapper procedure for the *Avg. daily infections* class. The right side of the graph shows the number dividing the feature values into low and high.

An example of such feature scores is shown in Figure 6. These results were obtained in 22 min with a Python implementation of the algorithm on a 3.79 GHz computer with 32 GB of RAM using 5 cores, one core for each cross-validation fold.

The pseudo-code of the Rule discovery algorithm is given in Algorithm 1.
**Algorithm 1** Pseudo-code of the Rule discovery algorithm.1:**for all** features **do**2: discretize feature3:**end for**4:**for all** folds in cross validation **do**5: generate all combinations of features and their discretized values6: each combination is one rule7: **for all** rules **do**8:  set rule decision as majority class of covered samples on training folds9:  on training folds calculate coverage, accuracy and rule score as multiplication of coverage and accuracy10:  **for all** features in rule **do**11:   calculate feature score as the rule score of rule with feature minus rule score of rule without feature12:  **end for**13:  **if** low coverage or low rule score or at least one feature with negative feature score **then**14:   discard rule15:  **end if**16: **end for**17: with respect to rule score select best 1000 rules per class18:**end for**19:**for all** distinct rules from all folds **do**20: assign union of covered training and test samples from all folds21: calculate coverage, accuracy, rule score, and feature score on union of test samples22:**end for**23:**for all** combinations of features and their discretized values **do**24: **for all** classes **do**25:   calculate average feature score as feature score multiplied by rule score over all the rules of the class26: **end for**27:**end for**28:**return** average feature score of each combination of features and their discretized values and classes

## 5. Results

### 5.1. Correlations between Classes

First, we calculated the Pearson correlations between the three picked classes. The *Avg. daily infections* class is not correlated to any other one, while the *Exponential* and *Reproductive rate* classes have a loose correlation of 0.42 (and are also more semantically similar to one another). All values are listed in Table 2. This result is significant as the *Avg. daily infections* and *Reproductive rate* metrics are the most popular in the related work, and the choice between these two metrics can significantly influence all achieved results.

Next, we show how the classes change if the time interval to calculate them is moved so that it starts with the countermeasures (Table 3). The effect on both the *Reproductive rate* and *Exponential* class is immediate—and most positive countries change their class. The *Avg. daily infections*, however, declines more slowly. We tried to move the time interval by another month into the future (relative to the already moved time interval), but almost half of the positive countries still remained positive.

The obvious conclusion is that the countermeasures taken were largely successful in limiting the infection rate—the number of infections quickly stopped growing, although it took some time for them to start falling again. A more important takeaway is how important it is to correctly acknowledge the countermeasures when selecting the data for analysis. If one is interested in features other than countermeasures, it probably makes sense to study the time period before they take effect.

### 5.2. Correlations between Features

We started with the list of features, split into different categories, as described in Section 3 (full list given in Table 4). Then, for each category, we eliminated every feature that had Pearson correlation with another in the same category higher than 0.8 or lower than −0.8. In the case of two features being correlated, the eliminated one was random. These features were not used in any of the following steps.

We chose not to remove features correlated with another feature in a different category as they could still offer a completely different explanation for the effect on the COVID-19 infection. For example, *Phones (per 1000)* and *Median age* have a high correlation (0.81), but one could indicate development and have an indirect relationship with COVID-19 infections, while the other could be directly indicating a potentially vulnerable demographic.

For example, looking at the *Health* category, we can observe that some diseases are highly correlated (they are not necessarily medically connected, but they are present in the same countries, e.g., due to the old population). If one of them is also correlated with the infection rate, it would be impossible to determine (from this data alone) which one, if any, is affecting the infection rate. This illustrates the benefit of having many features: even if one cannot identify the real cause, one at least has a comprehensive list of possible causes.

### 5.3. Significance of Individual Features

Feature significance in connection to COVID-19 was analyzed with both the statistical methods and ML (RF feature ranking) methods.

We first checked if the connection between the features and any of the classes is statistically significant. This experiment was repeated using *All*, *Selected*, and *Developed* country sets. The results can be found in Table 5.

The only features that were found significant on the selected set were nine from the *Avg. daily infections* class: *Individualism, Net migration, Plane passengers, Population, Tourists/population, GDP per capita (PPP), Phones (per 1000), Agriculture, Region: Western Europe, Tuberculosis immunization*. Additionally, there was one from the *Exponential* class: *Emotional stability*.

On the other hand, if looking at all countries, especially without the correction for multiple tests (using Bonferroni adjustment or similar), the majority (61 out of 93) of features were significant in regards to the *Avg. daily infections* class. Thus any research working with most countries is likely to find a connection between their chosen feature and class, despite the connection being indirect at best. This can explain the wide array of different results found in the literature.

The strong connection between most features and the infection rate, when all countries are used (set *All*), could stem from the fact that there is a strong correlation between the development of a country and its testing intensity—the correlation between countries chosen for this research based on testing and *Phones (per 1000)* feature is 0.50, while the same coefficient for the *Developed* feature is 0.45. Countries with no testing will clearly have the negative class and at the same time, have different feature values than those found in the developed countries.

In general, including in this experiment, the class *Avg. daily infections* tended to be the most correlated with the collected features and most predictable using ML methods (Table 6). Similarly, larger country sets were found more predictable than the smallest set that contains only the developed countries.

While it could be argued that having fewer instances in each subsequent country list automatically reduces the statistical significance of each feature, it should be noted that the set of best features is not only getting smaller but also different. Most of the features that have the biggest difference in *p*-values between the set of all countries and the set of selected countries come from the *Development* (8), followed by *Economy* (5) and *Weather* (4) categories. The full list of these features: *Temperature high, Wind gust, Climate, Openness, Power distance, Plane passengers, Tourists, Country prosperity score, Literacy, Infant mortality (per 1000 births), Judicial Effectiveness, Gov’t Spending, Service, Financial freedom, Trade freedom, Government efficiency, Tax burden % of GDP, Tariff rate (%), Inflation (%), Birthrate, Urban population (%), PM2.5, Diabetes, Respiratory disease, Cardiovascular disease, Smoking, Developed*.

As expected from the explanation in the previous paragraph, most of them can be linked to development. In fact, when development is controlled for (third column in Table 5), the remaining development indicators disappear. Notably, the significance of temperature is falling from each set to the subsequent one (the correlation coefficient between temperature and development is −0.58).

Next, we calculated the ten best features based on both the statistical significance (lowest *p*-values) and *RF feature ranking* for each class. This list was done only for the *Selected* set and is fully shown in Table 7. The statistical and ML methods agree on a good half of the selected features and overall have similar features in their respective top portions.

For the *Avg. daily infections* metric, the best features are either the indicators of mobility (*Plane passengers, tourists*, etc.) or development (*GDP per Capita (PPP), Agriculture*). Feature *Region_WESTERN EUROPE* is both an indicator of development and where the pandemic’s biggest early center was (aside from China).

The features chosen for the *Reproductive rate* and *Exponential* metrics are less clear. Many of them refer to the culture (*Social distance, Tightness, Emotional stability*) and others to health (*Vitamin D, Chronic kidney disease*), but it is harder to create a solid grouping.

### 5.4. Significance of Feature Groups

To understand if a particular category of features stands out, we tested their joint significance using the RF feature importance. The results for each category and each class can be found in the top section of Table 8.

Across different classes, the *Culture* and *Development* features seem to have the greatest impact, closely followed by the *Health* and *Travel* features. Other groups, however, are not trailing all that far behind. It seems that every feature category has its way of predicting the target class, which again could explain the diversity of the results found in the literature. The sole exception is the *Countermeasures* group, which only contains two features.

Another explanation for the significance of all the different feature categories is their interconnectedness. While some correlations were expected (e.g., *Economy* and *Development*), most feature groups seem to be heavily correlated, the exceptions being *Countermeasures* and *Travel*. Thus, if one of them shows a correlation with the COVID-19 infection, others are expected to as well. The full list of correlations between the categories is given in Table 9.

The interconnectedness of the feature categories could simply be a matter of us lacking the expert knowledge for grouping the features sensibly. So, in addition to the manually created categories, we tested automatically created feature clusters, created as described in Section 4.2.2 and Section 4.2.3. The same statistical tests were used for this as for individual features. The results are shown in Table 10.

It would be interesting to see if only a few such clusters would be significant against the COVID-19 class, as that could have helped us to isolate the relevant part of the feature space. However, that turned out not to be the case, with basically all feature clusters having a statistically significant correlation with the class—especially with the *Avg. daily infections* class.

The final test of feature categories was to use each of the manual categories individually in ML. RF was used for the task, using the LOIO procedure described in Section 4.3. The results are shown in the bottom section of Table 8. Compared to the RF score, *Culture*, *Travel*, and *Health* categories performed well. *Development* category under-performed, while *Geography* over-performed.

### 5.5. Machine Learning

In this section, we tried to create as good of ML models as possible on the available dataset. These models demonstrate that the information needed to predict the speed of COVID-19 spread is present in the data, even if it is not easy to determine what information precisely this is.

The ML procedure was again done using the LOIO method. The results for different classifiers and different classes are found in Table 11.

The Decision Tree and ADAboost classifiers performed best across the classes, although no classifier was best in all cases. The *Reproductive rate* had the lowest accuracy, hardly exceeding the baseline. The other two classes, however, could be predicted much better. Considering the lower baseline, the results for the *Avg. daily infections* stand out.

After FS using RF feature importance, few results changed, but the best accuracy achieved for each class remained similar. Both experiments, with and without FS, were repeated with no hyper-parameter tuning. However, there was no significant difference in the performance so we can conclude that tuning was not a crucial part of our procedure.

Then, we tried the Wrapper FS technique described in section *Feature selection with a Wrapper method*. Using this method and the three best classifiers from the previous experiment, we achieved the results in Table 12. The accuracy was significantly improved in all cases. The experiment was repeated using the Area Under Curve (AUC) score instead of accuracy, and the results are shown in Table 13. The classifier’s relative score remained mostly the same, with RF becoming slightly better relative to the other two classifiers. Finally, we tried Boruta FS, which resulted in very similar features and only slightly worse accuracy than the Wrapper method. Therefore, we chose the Wrapper method, but given that the difference is not statistically significant, we deem Boruta FS appropriate for this domain as well.

Finally, we compared the predictability of different country sets. Larger sets (e.g., *All*) seem easier to classify with a good accuracy. A question that remained is whether the extra difficulty is merely due to the size of the sets.

We created two additional sets to answer the question: the *Semi-selected* set is of the same size as the *Selected*, but only half its countries are from the *Selected* set. The same relation holds between the *Developed* and *Semi-developed* set. The accuracy on these two sets, however, is much larger than the one on their same-size counterparts (full list of results in Table 6). This could be explained by having more countries that are less developed in these mixed sets (mostly belonging to the negative class, possibly due to insufficient testing). These countries can be classified using the many *Development* and correlated features, which makes classification easier.

## 6. Feature Discussion

In this work, we evaluated the significance of features with three metrics: statistical tests, RF feature importance, and the Wrapper method. The features selected by the three metrics were quite similar, but they were substantially different when compared across the three different classes. In this section, we focus on the *Avg. daily infections* class (as it has the strongest correlation with the features) and the Wrapper method (as it produces the most diverse and least redundant features). The features selected this way are listed in Table 14. For each, we also list the group of correlated features (correlation coefficient > 0.65). To observe if the values of these features promote or inhibit the spread of infection, we generated classification rules and then evaluated the contributions these values, discretized into low and high, to the negative and positive class (slow and fast spread). The results are shown in Figure 6. For example, the blue score of low GDP ($ per capita) means that including low GDP ($ per capita) moderately improves the accuracy of rules predicting slow spread. The orange score of high GDP ($ per capita) means that including high GDP ($ per capita) substantially improves the rules predicting fast spread. We also briefly discuss features found significant according to the other metrics and classes—the full list is provided in Table 7 and their individual contributions in Figure 6.

The largest feature group is the one containing the *GDP per capita* feature and the ones correlated with it. They all indicate development, but as discussed before, development itself cannot be the direct cause of the fast spread of the infection. The two features in this group that could be more relevant are *Net migration* and *Agriculture*. The *Agriculture* feature may be an indirect indicator of population density, and the agricultural labor environment is probably also less conductive to infection spread than industrial and service. Another common-sense factor that contributes to the infection spread and is connected to development is traveling. The *Net migration* is about permanent migration, so it is only implicitly connected to travel. However, features like *Plane passengers/population* and *Tourists/population* were considered among the most statistically significant, but they are not on the Wrapper list because they were considered redundant by the classifier when *GDP per capita* was included. Looking at other metrics of feature significance across all three classes, quite a few features from the *Development* and *Economy* categories were found significant, although no single feature stands out (probably due to how interconnected and thus interchangeable they are). The correlation of COVID-19 infections with the features of this type matches the results in the related work [6,16].

There are three health-related features in Table 14, which makes this category the best represented: *Blood 0*, *Vitamin D*, and *Tuberculosis immunization*. The first two are particularly interesting as they have no feature strongly correlated with them, and they were also shown to be relevant in the related work. The nature of their relation with the class is the opposite of what was found in the related work, though: larger frequency of the blood 0 group seems to promote the infection, while vitamin D deficiency inhibits it. The former could be true, but since this feature was only significant for the *Avg. daily infections* class and Wrapper FS, it could also be a fluke. The unexpected finding regarding vitamin D deficiency is almost certainly a fluke, since it is quite weak, and we found the opposite for the classes other than *Avg. daily infections*: vitamin D deficiency promoted the infection there, and the effect was also considerably stronger. The impact of *Tuberculosis immunization* is clear and matches the results in the related work [66]: low immunization contributes to COVID-19 infection. Since the feature is correlated with development features (immunization is more common in developed countries, which tend to be more infected), it means this feature is not merely an indirect indicator of development. Finally, *Chronic kidney disease* was found significant for classes other than *Avg. daily infections*.

Table 14 contains two features from the *Culture* category: Openness and Individualism. Countries with high scores in Individualism have faster infection spread, which can be explained by their citizens being less loyal and supportive of their fellow people and also caring less about their actions that could possibly hurt others (e.g., being careful regarding the spread of infections). Countries with high scores in *Openness* also correlate with faster infection spread. The possible explanation for this is less clear than the previous one, but people with high scores in openness show more anti-authority tendencies. This could therefore result in individuals in countries with high scores in Openness disregarding governmental recommendations on behavior that prevents infection spreading due to the source of these recommendations. The strong negative correlation of this feature with *Region: Asia (ex. near east)* suggests that such tendencies are not common in Asia. In general, features from the *Culture* category are the most represented among significant features for all metrics and classes. It is of note that these features are not analyzed in such detail in any other related work we are aware of.

The *PM2.5* and *Region: Western Europe* features may just be development indicators, with the latter also indicating one of biggest early COVID-19 epicenters. One would expect (and as found in some related work) that the PM2.5 concentration—which is harmful to the lungs—would contribute to COVID-19 infection, but the reverse was found to be true. This is probably because more developed countries have less polluting industries and cleaner air. Countries with larger *Population* may exhibit slower infection spread because the infection starts in a limited number of places where it was introduced into the country, and it takes more time to spread throughout a larger country. Finally, the *COVID Awareness* feature is related to faster infection spread, so it appears not to have a preventive effect but is rather a natural consequence of greater infection. The *PM2.5*, *Region: Western Europe* and *Population* features are also significant according to some other metrics and classes, while *COVID Awareness* is exclusive to the *Avg. daily infections* and Wrapper combination.

### Limitations of the Study

The main limitation of the study is that we use only a few months of COVID infection data out of over a year. This choice was, however, deliberate: doing so can remove the impact of countermeasures when studying the impact of other factors. Analyzing the impact of countermeasures was out of the scope for this paper and was done separately in our other work [76].

Another limitation was our definition of binary classes. They were chosen in order to increase the robustness and reliability of our work, but the fact that they were not standard made direct comparisons with the related work more difficult.

Considering only infections and not deaths, whose data may be more reliable, is also a limitation. We decided to focus on infections because this is the variable that receives the most attention and most strongly shapes pandemic-related policies across the globe.

Lastly, our findings cannot conclusively determine the causal nature of any of the studied features, and we believe that such conclusions cannot be drawn using ML alone. However, ML can point to likely contributors to fast infection spread, and we believe this study has done so the most comprehensively to date.

## 7. Conclusions

The goal of this work was to determine which factors—excluding countermeasures—contribute the most to the virus’s fast spread. The related work applying statistical and ML methods on early COVID-19 data, however, points to different, sometimes conflicting, contributing factors.

We attempted to remedy the weaknesses identified in the approaches proposed in the related work: our main methodological contribution was the careful selection of countries and time periods for analysis, which was often not done in related work. First, we showed the importance of selecting only countries with adequate testing. If this or a similar step is not done, the number of significant features rapidly increases. However, these features—many of which are related to development, as developed countries tested more—probably only indicate that one finds more infections when tests for them more. Second, not accounting for the beginning of the countermeasures can completely change some of the infection metrics (in our example, all positive instances for the *Reproduction rate* class changed). We also developed a new rule discovery algorithm adapted to extracting all possible relations from data and presenting them in a manner understandable to humans. Most related work used various regression methods, which are also understandable but cannot capture all the relations our rules can. In the process, we collected the largest dataset of different, relevant features, some of which were not found in similar studies (to the best of our knowledge). This dataset is publicly available at [34].

The analysis of feature categories showed that the most significant ones are *Culture* and *Development*, followed by *Travel* and *Health*. This suggests that the most vulnerable regions are developed ones with good connections to the rest of the world, preferably with a culture that does not promote values that result in following recommended behavior for preventing COVID-19 spread. The finding about culture is particularly interesting as culture was not much researched in related work. Despite studying many development-related features, it remains unclear which factors exactly made developed countries particularly susceptible to COVID-19. A possible explanation is that general development features characterize countries with multiple relevant factors, some of which were also identified as relevant on their own (e.g., culture and travel), while others are not strong enough on their own but also make a contribution (e.g., population density). The *Weather* category—which was probably the one most studied in related work—did not stand out as especially important.

Turning to individual features, we used our rule-discovery algorithm to identify a few that deserve particular attention. Of the cultural features, *Individualism* and *Openness*—possibly due to anti-authority tendencies—are the most prominent and can guide the development of countermeasures, especially “soft” ones such as publicity campaigns. The finding regarding the preventive effect of tuberculosis immunization corroborates the related work, but we would argue that the fact it stood out from a very large set of features in carefully selected countries is a stronger finding. Since it can be acted upon, we believe it deserves further investigation. Some developed countries discontinued tuberculosis immunization because of a decrease in tuberculosis incidence, but if the vaccine has benefits beyond tuberculosis, the decision may be re-evaluated.

An obvious direction for future research is studying the impact of countermeasures and their interplay with the factors investigated in this paper. We have done some preliminary research, which suggested that countermeasures indeed overshadow other factors. However, some related work disagrees [23], so the problem appears to be a complex one. Another direction is to investigate ways to increase the reliability of infection data, possibly by combining infections, deaths, and testing rate. These variables are clearly related, but the exact relation is difficult to establish, mainly because the number of all infections can only be inferred from antibody tests, and such data is scarce. Additional features and machine-learning methods could also be investigated, although we do not foresee a large benefit unless some important insight about relevant features is obtained beforehand.

In summary, we found that our infection metrics can be predicted from the features data reasonably well, with an accuracy of up to 86%, compared to the 50–60% baseline. There is no single feature in our list that would decisively impact the infection spread, but we did uncover some interesting ones. The less than perfect accuracy suggests that there must either be additional factors that affect the spread of COVID-19 or a part of the error is due to chance, for example, which countries the first infected tourists traveled to.

## Figures and Tables

**Table 1:** The proportion [%] of instances considered positive for each class/country set combination. In addition, the number of countries (and thus the number of instances) in each set is also listed.

	All	Selected	Developed	Semi-Selected	Semi-Developed
Daily avg.	23	49	71	30	50
Repr. rate	30	61	46	30	64
Exponential	39	68	64	35	64
# Countries	149	59	35	60	36

**Table 2:** Correlation between the three classes (binarized infection metrics).

	Avg. Daily	Repr. Rate	Exponential
Avg. daily	1	0.12	0.12
Repr. rate	0.12	1	0.42
Exponential	0.12	0.42	1

**Table 3:** The number of countries that are classified positive according to one of the infection classes. We compare the time interval before countermeasures (that we used) to a time interval of the same length starting with the countermeasures. In the [ext] row, the time interval was moved forward by another month.

	Number of Positive Countries	
	Before	After
Repr. rate	36	0
Exponential	40	12
Avg. daily	29	23
Avg. daily [ext]	29	17

**Table 4:** All features, divided into different categories. Features highly correlated inside their group are grayed out. The number of features for each category is given in brackets.

Category	Features
Weather (10)	Temperature high, Humidity, Pressure, Wind gust, Cloud cover, Precip. intensity, Precip probability, Visibility, Climate, PM2.5, Temperature Low, Temperature max, Temperature min, Apparent temperature high, Apparent temperature low, Apparent temperature max, Apparent temperature min, Dew point, Ozone, Wind speed, UV Index, Precip. intensity max
Culture (14)	Extraversion, Emotional stability, Agreeableness, Conscientiousness, Openness, Power distance, Individualism, Masculinity, Uncertainty avoidance, Future orientation, Indulgence, Social distance, Tightness, Completed tightness
Travel (8)	Plane passengers, Tourists, Net migration, Plane passengers/population, Tourists/population, Mobility-driving, Mobility-walking, FDI/GDP, Tourists normalized, FDI Inflow (Millions), Plane passengers normalized
Health (11)	Diabetes, Respiratory disease, Cardiovascular disease, Obesity, Smoking, ACE II, Blood O, Chronic kidney disease, Vitamin D, Tuberculosis immunization, Death rate, Dementia, Cancer, Median age, Birthrate
Economy (13)	GDP (Billions, PPP), GDP per capita (PPP), GDP growth rate (%), 5 year GDP growth rate (%), Unemployment (%), Tax burden, Tax burden % of GDP, Tariff rate (%), Corporate tax rate (%), Public debt (% of GDP), Fiscal health, Inflation (%), Gov’t spending, GDP ($ per capita)’, Income tax rate (%)
Development (15)	Developed, Country prosperity score, Region prosperity score, Phones (per 1000), Literacy (%), Infant mortality (per 1000 births), Agriculture, Industry, Service, Monetary freedom, Labor freedom, Financial freedom, Trade freedom, Government efficiency, World rank, Property rights, Business freedom, Judicial effectiveness, Government integrity, Government effectiveness index, Education
Geography (19)	Population, Area (sq. mi.), Pop. Density (per sq. mi.), Coastline (coast/area ratio), Arable (%), Crops (%), Urban population (%), Region: Asia (ex. near east), Region: Baltics, Region: C.W. of Ind. states, Region: Eastern Europe, Region: Latin Amer. Carib, Region: Near East, Region: Northern Africa, Region: Northern America, Region: Oceania, Region: Sub-Saharan Africa, Region: Western Europe, ‘Other (%)’
Countermesures (2)	Eventual countermeasures, COVID awareness

**Table 5:** The number of features that are statistically significant based on the class, dataset, and whether the correction, using Bonferroni adjustment (marked “Corrected”), is used or not—see Section 4.1.1.

	All		Selected		Developed	
	Base	Corrected	Base	Corrected	Base	Corrected
Avg. daily	61	37	40	9	7	0
Repr. rate	48	31	4	0	6	0
Exponential	26	5	26	1	4	0

**Table 6:** Accuracy (%) achieved by the AdaBoost model, which often (but not always) performed the best for different data subsets (All, Selected, Developed, Semi-selected, Semi-developed).

	All	Selected	Developed	Semi-Selected	Semi-Developed
Avg. daily	85	76	68	87	89
Repr. rate	74	68	46	77	46
Exponential	63	66	39	62	71

**Table 7:** Features that were in the top list for at least one class and feature ranking combinations. Such combinations are explicitly marked. Feature rankings are marked: ST—using statistical methods, RF—Using the Random Forest classifier, and WR—using the Wrapper FS procedure.

	Avg. Daily			Repr. Rate			Exponential		
**Weather**	ST	RF	WR	ST	RF	WR	ST	RF	WR
Temperature high						✓			
PM2.5	✓	✓	✓						
**Culture**	ST	RF	WR	ST	RF	WR	ST	RF	WR
Extraversion							✓		✓
Emotional stability							✓	✓	✓
Agreeableness								✓	
Conscientiousness								✓	✓
Openness		✓	✓	✓	✓	✓			
Power distance							✓	✓	✓
Individualism	✓	✓	✓						
Social distance								✓	✓
Tightness				✓	✓	✓			
**Travel**	ST	RF	WR	ST	RF	WR	ST	RF	WR
Net migration	✓	✓							
Plane passengers/pop.	✓	✓							
Tourists/pop.	✓	✓							
Mobility-driving						✓			
Mobility-walking					✓				
Plane passengers norm.								✓	✓
**Health**	ST	RF	WR	ST	RF	WR	ST	RF	WR
Blood 0			✓						
Chronic kidney disease						✓	✓	✓	✓
Vitamin D			✓	✓	✓	✓		✓	✓
Tuberculosis immun.	✓		✓						
**Economy**	ST	RF	WR	ST	RF	WR	ST	RF	WR
GDP per capita (PPP)	✓	✓	✓						
Unemployment (%)				✓	✓	✓			✓
Tax burden				✓	✓	✓			
Tariff rate (%)					✓				
Fiscal health							✓	✓	✓
Gov’t spending				✓	✓				
**Development**	ST	RF	WR	ST	RF	WR	ST	RF	WR
Developed							✓		
Country prosperity							✓		
Phones (per 1000)	✓						✓	✓	
Agriculture	✓								
Industry						✓			
Trade freedom							✓		
Government efficiency					✓				
Judical effectiveness		✓							
**Geography**	ST	RF	WR	ST	RF	WR	ST	RF	WR
Population		✓		✓					
Coastline (coast/area)				✓		✓			
Region: Western Europe	✓		✓	✓					
**Countermeasures**	ST	RF	WR	ST	RF	WR	ST	RF	WR
Eventual counterm.				✓					
COVID awareness			✓						

**Table 8:** **Top**: Aggregate feature ranking using RF feature score. These values are normalized so that they sum to 1. **Bottom**: Accuracy (%) when using only one feature category when training and testing the classifier.

**RF Feature Score**	**Daily Average**	**Reproduction Rate**	**Exponential**	**Average**
Weather	0.09	0.09	0.08	0.09
Culture	0.14	0.18	0.21	0.18
Travel	0.18	0.12	0.08	0.13
Economy	0.09	0.15	0.13	0.12
Development	0.12	0.16	0.18	0.18
Geography	0.11	0.12	0.06	0.10
Health	0.11	0.11	0.19	0.14
Countermeasures	0.06	0.04	0.02	0.04
**Accuracy**	**Daily Average**	**Reproduction Rate**	**Exponential**	**Average**
Weather	71	54	54	60
Culture	74	61	73	69
Travel	74	69	68	70
Economy	61	59	68	63
Development	68	52	59	60
Geography	78	63	58	66
Countermeasures	59	58	58	58
Health	75	56	59	63
Baseline	49	61	67	59

**Table 9:** Correlation between different feature categories. Each category was summarized by the first PCA component of all the features in the category, and correlations between these components were taken as the proxy for the correlation between the categories.

	Weat.	Cult.	Trav.	Econ.	Devel.	Geo.	Count.	Health
Weat.	1.0	0.35	0.22	0.59	0.56	0.52	0.13	0.58
Cult.	0.35	1.0	0.23	0.54	0.64	0.55	0.01	0.62
Trav.	0.22	0.23	1.0	0.12	0.16	0.19	0.04	0.12
Econ.	0.59	0.54	0.12	1.0	0.70	0.80	0.08	0.77
Devel.	0.56	0.64	0.16	0.70	1.0	0.65	0.06	0.87
Geo.	0.52	0.55	0.19	0.80	0.65	1.0	0.20	0.75
Count.	0.13	0.01	0.04	0.08	0.06	0.20	1.0	0.02
Health	0.58	0.62	0.12	0.77	0.87	0.75	0.02	1.0

**Table 10:** Statistical significance of different feature groupings. The groupings were done either manually or with K-means or hierarchical clustering ($H_{c}$—using absolute correlation as distance, $H_{t}$—using Euclidian distance between values of different feature values directly). The label *B* stands for the base statistical test, while *C* for the test with the applied correction.

	Manual		KMeans		$H_{c}$		$H_{t}$	
Cluster number	9		4		8		8	
**Test type**	**B**	**C**	**B**	**C**	**B**	**C**	**B**	**C**
Avg. daily	7	5	4	3	7	7	6	5
Repr. rate	2	0	0	0	0	0	0	0
Exponential	5	0	4	0	8	1	4	0

**Table 11:** The accuracy (%) achieved for different ML algorithms with hyper-parameter tuning and all features. The experiment was repeated for each class, and the highest accuracy is shown in bold.

	Baseline	kNN	DT	RF	XGB	SVC	ADA
Avg. daily	51	69	**76**	**76**	71	63	**76**
Repr. rate	61	61	59	61	61	61	**63**
Exponential	68	58	**76**	73	68	59	69

**Table 12:** The accuracy (%) achieved for different ML algorithms with hyper-parameter tuning and features selected by the Wrapper procedure. The experiment was repeated for each class, and the highest accuracy is shown in bold.

	Baseline	DT	RF	ADA
Avg. daily	51	**86**	80	83
Repr. rate	61	**71**	70	66
Exponential	68	66	**70**	**70**

**Table 13:** The Area Under Curve (AUC) score (%) achieved for different ML algorithms with hyper-parameter tuning and features selected by the Wrapper procedure. The experiment was repeated for each class, and the highest accuracy is shown in bold.

	DT	RF	ADA
Avg. daily	73	**86**	81
Repr. rate	72	75	**78**
Exponential	74	**78**	73

**Table 14:** Features selected using the Wrapper procedure for the *Avg. daily infections* class, alongside their category and the list of correlated features.

Feature	Category	Correlated
GDP per Capita (PPP)	Development	Net migration, Developed, Agriculture, Government efficiency, Phones (per 1000), Judical effectiveness, GDP ($ per capita), Government integrity, Property rights
Blood O	Health	/
Tuberculosis immunization	Health	GDP ($ per capita), Individualism, Developed, Phones (per 1000), Power distance, Net migration
Vitamin D	Health	/
Openness	Culture	Region: Asia (ex. near east), Tax burden % of GDP
Individualism	Culture	Power distance, Cancer
PM2.5	Weather	Literacy (%)
Population	Geography	/
Region: Western Europe	Geography	Developed, Tax Burden, Gov’t Spending, Respiratory disease, GDP ($ per capita)
COVID Awareness	Countermeas.	/

## Data Availability

All the data and code used is available at [34]. External sources used to build our dataset are listed in Section 3.3.

## Abbreviations

The following abbreviations are used in this manuscript:

ML	Machine Learning
RF	Random Forest
FS	Feature Selection

## Appendix A

In this appendix we list the feature categories, automatically generated by clustering algorithms in Section 4.2. The K-means clustering algorithm found 4 clusters that are listed in Table A1, while the hierarchical clustering generated the dendrogram seen in Figure A1. It should be noted that in the case of very heavily correlated features (as indicated in Table 4) only one is shown in this appendix for clarity. They would, of course, all go into the same clusters.

**Table A1:** Features, divided into four categories using K-means clustering.

Clusters	Features
Cluster 1	Temperature high, Pressure, Visibility, Emotional stability, Power distance, Masculinity, Uncertainty avoidance, Social distance, Tightness, Plane passengers normalized, GDP growth rate (%), 5 Year GDP growth rate (%), Govt spending, Tax burden, Corporate tax rate (%), Vitamin D, BCG adjusted
Cluster 2	Wind gust, Precip probability, Climate, Conscientiousness, Individualism, Future orientation, Net migration, Mobility-walking, Region prosperity score, GDP per capita (PPP), Phones (per 1000), Tax burden % of GDP, Arable (%), Death rate, Region: Western Europe, Diabetes, Respiratory disease, Cardiovascular disease, ACE II, Chronic kidney disease
Cluster 3	Humidity, Cloud cover, Extraversion, Agreeableness, Openness, Indulgence, Country prosperity score, Literacy (%), Judicial effectiveness, Service, Monetary freedom, Labor freedom, Financial freedom, Trade freedom, Government efficiency, Fiscal health, Urban population (%), Obesity
Cluster 4	Precip. intensity, Google Trends, Plane passengers, Tourists, Plane passengers/population, Tourists/population, Mobility-driving, GDP (Billions, PPP), Unemployment (%), FDI Inflow (Millions), Infant mortality (per 1000 births), Agriculture, Industry, Tariff rate (%), Public debt (% of GDP), Inflation (%), Population, Area (sq. mi.), Pop. Density (per sq. mi.), Coastline (coast/area ratio), Crops (%), Birthrate, PM2.5, Region: Asia (ex. near east), Region: Baltics, Region: C.W. of Ind. states, Region: Eastern Europe, Region: Latin Amer. Carib, Region: Near East, Region: Northern Africa, Region: Northern America, Region: Oceania, Region: Sub-Saharan Africa, Smoking, Blood O
